# Blood-Based Biomarkers for Traumatic Brain Injury: A New Era in Diagnosis and Prognosis

**DOI:** 10.3390/ijms262412158

**Published:** 2025-12-18

**Authors:** Giulia Pignataro, Marta Sacco Fernandez, Marcello Candelli, Gloria Rozzi, Andrea Piccioni, Evelina Forte, Francesco Franceschi

**Affiliations:** 1 Department of Emergency, Anesthesiological and Reanimation Sciences, Fondazione Policlinico Universitario A. Gemelli–IRCCS, 00168 Rome, Italy; marcello.candelli@policlinicogemelli.it (M.C.); gloria.rozzi01@icatt.it (G.R.); andrea.piccioni@policlinicogemelli.it (A.P.); evelina.forte@policlinicogemelli.it (E.F.); francesco.franceschi@policlinicogemelli.it (F.F.); 2 Department of Translational Medicine and Surgery, Università Cattolica del Sacro Cuore, 00168 Rome, Italy; marta.saccofernandez01@icatt.it

**Keywords:** traumatic brain injury clinical features, traumatic brain injury pathophysiology, TBI and dementia, traumatic brain injury biomarkers, mTBI biomarkers, GFAP and TBI, UCH-L and traumatic brain injury, prognostic biomarkers in TBI, TBI and inflammation

## Abstract

Traumatic brain injury (TBI) is a major global health concern and a leading cause of mortality and disability. Head computed tomography (CT) remains indispensable for the detection of intracranial hemorrhage; however, its indiscriminate use in mild trauma increases radiation exposure, cumulative oncogenic risk, and healthcare costs. Consequently, there is growing interest in tools capable of improving sensitivity in mild or early-stage TBI. Protein-based biomarkers are promising complements to conventional assessment. Molecules such as glial fibrillary acidic protein (GFAP), ubiquitin C-terminal hydrolase L1 (UCH-L1), S100 calcium-binding protein B (S100B), and neurofilament light chain (NfL) reflect astroglial activation, neuronal injury, and axonal damage, enabling objective evaluation of neurotrauma. Beyond protein biomarkers, metabolomic and lipidomic approaches capture alterations associated with early metabolic distress, oxidative stress, mitochondrial dysfunction, and membrane disruption following TBI. High-resolution mass spectrometry studies have identified reproducible metabolite and lipid signatures correlating with injury severity and functional outcomes. Longitudinal profiling further reveals dynamic metabolic trajectories that distinguish secondary injury progression from stabilization, supporting predictive modeling and risk stratification. Together, these advances pave the way toward precision medicine in neurotrauma. Nevertheless, variability in assay performance and sampling timing continues to limit widespread clinical adoption. Future research should prioritize methodological standardization, analytical validation, and the integration of multi-omic data with machine learning–based predictive models.

## 1. Introduction

Traumatic brain injury (TBI) represents a major global public health challenge, affecting approximately 50 million individuals each year and ranking among the leading causes of mortality and long-term disability across all age groups. The associated economic burden is considerable, with global costs estimated to exceed USD 400 billion annually, encompassing both direct healthcare expenditures and indirect losses related to reduced productivity and long-term disability [1]. TBI most frequently arises from external mechanical forces, including road traffic accidents, falls, sports-related injuries, and blast exposures, resulting in a wide and heterogeneous spectrum of neurological impairments. Falls are particularly prevalent among the elderly and very young, whereas road traffic accidents constitute the predominant cause in younger adult populations. In military and conflict settings, blast-related injuries represent a major etiological factor, as blast waves generate a combination of primary overpressure effects and rapid acceleration–deceleration forces, leading to TBI that may range from mild concussion to severe and disabling neurological damage [2,3]. TBI is an intrinsically heterogeneous disorder, traditionally classified according to injury severity (mild, moderate, or severe), underlying mechanism (focal versus diffuse injury), and clinical presentation. This marked heterogeneity poses significant challenges to accurate diagnosis, prognostication, and the development of individualized treatment strategies. In addition, TBI is frequently associated with multisystem consequences that extend beyond the central nervous system, thereby requiring a structured, multidisciplinary approach to clinical management. Among the most common post-traumatic complications, headache represents a major source of morbidity, often manifesting with migraine-like or tension-type features that substantially impair quality of life [4,5]. Notably, even mild TBI can disrupt migraine-related neurobiological pathways, leading to increased frequency and severity of headache attacks [6]. Visual disturbances are also frequently reported, including oculomotor dysfunction, accommodative deficits, and impairments in higher-order visual processing [7]. Taken together, these manifestations underscore the complex cascade of neurophysiological and sensory alterations triggered by TBI, which extend well beyond the initial mechanical insult and exert a sustained impact on cognitive, sensory, and psychosocial functioning. While moderate to severe TBI is often characterized by evident structural abnormalities on neuroimaging, the nature and extent of intracranial lesions are closely associated with the need for neurointensive interventions and represent key predictors of clinical outcomes [8]. In contrast, mild TBI (mTBI) frequently presents a diagnostic challenge, as conventional neuroimaging may appear normal despite the persistence of cognitive, emotional, or behavioral symptoms [9]. To address these limitations, the recently proposed National Institute of Health–National Institute of Neurological Disorders and Stroke (NIH–NINDS) classification of acute TBI introduces a standardized framework that integrates measurable biological, clinical, and imaging parameters, moving beyond traditional severity-based scoring systems. This novel approach is designed to capture the full spectrum of injury mechanisms and patient phenotypes, thereby enabling more precise diagnosis, improving comparability across research studies, and supporting the development of personalized therapeutic strategies in diverse clinical settings [10]. In patients with moderate to severe TBI, updated prognostic criteria further provide intensive care clinicians with a more robust, evidence-based framework for clinical decision-making, enhancing the prediction of neurological outcomes and the tailoring of therapeutic interventions [11].

### Pathophysiological Mechanisms

Conventional diagnostic tools, including the Glasgow Coma Scale (GCS) and neuroimaging, remain fundamental in the acute management of TBI; however, their sensitivity is limited, particularly in mild or early-stage injuries and in elderly patients [12]. These limitations underscore the need for more sensitive and objective approaches capable of monitoring disease evolution and identifying potential therapeutic targets. As a result, there has been growing interest in the application of biomarkers [13]—objective and quantifiable biological indicators that can complement traditional clinical assessments and offer mechanistic insights into neuronal and axonal injury, astroglial activation, and neuroinflammatory processes. TBI is characterized by a biphasic injury process consisting of an initial primary insult, which occurs at the moment of impact, and a secondary injury phase that evolves over minutes to days—or even longer—through a complex cascade of cellular and molecular events [14]. The primary injury is driven by mechanical forces such as direct impact, rapid acceleration–deceleration, blast exposure, or penetrating trauma, leading to focal lesions (including contusions and hematomas), diffuse axonal injury, and vascular disruption [15]. Diffuse axonal injury is particularly prevalent in moderate to severe TBI and results from shearing and stretching of axons within white matter tracts. Although frequently undetectable on conventional neuroimaging during the acute phase, diffuse axonal injury is strongly associated with persistent cognitive, behavioral, and motor impairments [16]. In the aftermath of the primary insult, a multifaceted cascade of secondary pathophysiological mechanisms is initiated, encompassing glutamate-mediated excitotoxicity, intracellular calcium overload, mitochondrial dysfunction, oxidative stress, neuroinflammation, and apoptosis [15,17]. Activation of microglia and reactive astrocytosis exert context-dependent effects that may be either neuroprotective or neurotoxic, depending on the timing, magnitude, and duration of the response [18]. Concurrently, disruption of the blood–brain barrier facilitates the extravasation of plasma proteins, promotes cerebral edema, and allows for infiltration of peripheral immune cells, thereby amplifying local tissue damage [19]. Proinflammatory cytokines, including IL-1β, tumor necrosis factor- α (TNF-α) and interleukin-6 (IL-6), play central roles in modulating neuronal survival and glial activation. When sustained, this inflammatory milieu may drive progressive neurodegeneration, providing a mechanistic link between TBI and the later development of neurodegenerative conditions such as Alzheimer’s disease, chronic traumatic encephalopathy, and Parkinsonian syndromes [20,21]. These metabolic and inflammatory consequences not only mediate secondary brain injury but also promote the release of brain-derived molecules into the bloodstream or cerebrospinal fluid, thereby providing the biological foundation for biomarker detection. Recent studies have identified several promising protein-based biomarkers, including glial fibrillary acidic protein (GFAP), ubiquitin carboxy-terminal hydrolase L1 (UCH-L1), S100 calcium-binding protein B (S100B), tau, neurofilament light chain (NfL), and neuron-specific enolase (NSE). These molecules, measurable in blood or cerebrospinal fluid, exhibit substantial potential not only for diagnostic purposes but also for prognostic assessment, therapeutic monitoring, and patient risk stratification [22,23,24,25,26,27]. The interpretation of circulating biomarkers in TBI is profoundly influenced by the marked etiological heterogeneity of the condition, as well as by several additional confounding factors that require careful consideration. Age represents a key determinant of baseline biomarker concentrations and post-injury kinetics, particularly for proteins associated with neurodegeneration and axonal injury [4]. Likewise, both the mechanism and the magnitude of the traumatic insult—such as blast versus blunt trauma, or rotational versus predominantly linear acceleration—critically modulate the pattern and extent of neuronal, axonal, and glial damage, thereby generating distinct biomarker release profiles. In addition, extracranial injuries, especially those involving skeletal muscle, bone, or peripheral nerves, may substantially contribute to circulating biomarker levels, ultimately reducing their specificity for central nervous system (CNS) injury. Finally, the profound pathophysiological complexity of TBI—including the coexistence of focal and diffuse lesions, the interaction between primary and secondary injury cascades, and the dynamic temporal evolution of tissue damage—poses a significant challenge to biomarker standardization and limits their immediate translation into routine clinical practice [4]. This review aims to provide a comprehensive overview of the role of biomarkers in TBI, emphasizing their biological significance, clinical utility, existing limitations, and future directions for research and clinical implementation.

## 2. Classification of Biomarkers in TBI

Biomarkers in TBI have become a cornerstone of contemporary neurotrauma research, providing objective and quantifiable indicators of neuronal and glial injury. Their clinical and scientific relevance encompasses the entire continuum of TBI management—from early diagnosis and prognostication [28] to longitudinal monitoring and evaluation of therapeutic efficacy [29,30]. By complementing conventional clinical assessment and neuroimaging, biomarkers introduce a vital dimension of biological specificity, enabling more accurate risk stratification, supporting personalized therapeutic strategies, and deepening the overall understanding of TBI pathophysiology [31]. Among the most extensively validated protein biomarkers are GFAP and UCH-L1 [32]. GFAP, an intermediate filament protein localized within astrocytes, is released into the bloodstream following astroglial injury and disruption of the blood–brain barrier [33]. UCH-L1, a neuronal enzyme involved in ubiquitin-dependent protein turnover and synaptic maintenance, serves as a sensitive marker of neuronal cell body injury [34]. The combined measurement of GFAP and UCH-L1 provides complementary insights into glial and neuronal damage and forms the basis of the Banyan Brain Trauma Indicator™, the first FDA-approved in vitro diagnostic test for TBI. This biomarker panel has demonstrated high diagnostic accuracy (AUC > 0.93) in detecting CT-positive intracranial lesions in mild TBI, outperforming earlier candidates such as S100B [35]. By minimizing unnecessary neuroimaging and identifying patients at risk for clinically significant lesions, these assays mark a pivotal advance toward the integration of molecular diagnostics into the acute evaluation of head injury.

### 2.1. Diagnostic Role

In the acute phase of TBI, diagnostic biomarkers offer rapid and objective indicators of structural and cellular injury. Their principal clinical value lies in enabling early diagnosis, optimizing triage decisions, and minimizing unnecessary neuroimaging—particularly in mild cases, where conventional imaging often fails to detect overt structural abnormalities [29,36]. Both GFAP and UCH-L1 exhibit distinct temporal release profiles: UCH-L1 levels rise sharply within the first six hours after injury and return to baseline within approximately 24 h, whereas GFAP concentrations increase more gradually and remain elevated for up to 72 h [37]. Elevated levels of either marker are strongly associated with the presence of intracranial lesions on CT, while low concentrations reliably predict their absence. Consequently, the combined assessment of GFAP and UCH-L1 enables clinicians to safely identify patients unlikely to benefit from head CT, thereby reducing unnecessary imaging in the evaluation of mild TBI [38]. Large multicentre studies, including TRACK-TBI and CENTER-TBI, have demonstrated that the combined measurement of GFAP and UCH-L1 achieves sensitivities exceeding 97% and specificities approaching 99% for detecting CT-positive lesions within the first 12 h post-injury [35]. These findings support the use of serum biomarkers as an effective adjunct to conventional diagnostic protocols, particularly in emergency and prehospital settings. Additional complementary biomarkers—such as S100B, NfL, and tau protein—reflect astrocytic and axonal injury and may further enhance diagnostic precision when incorporated into multimarker algorithms. This biochemical CT-equivalent triage strategy represents a promising approach to optimize resource utilization, minimize radiation exposure, and reduce healthcare costs while maintaining diagnostic safety and accuracy in the acute management of TBI [39]. The diagnostic accuracy of these biomarkers is summarized in Table 1, Table 2, Table 3, Table 4, Table 5 and Table 6.

### 2.2. Prognostic Role

Beyond their diagnostic utility, biomarkers have also demonstrated substantial prognostic value in predicting clinical outcomes and recovery trajectories following TBI [27,40]. Both GFAP and UCH-L1 show strong correlations with mortality and functional outcomes at six months, as consistently reported in the TRACK-TBI and CENTER-TBI cohorts. Elevated concentrations of these biomarkers within the first 24 h post-injury are predictive of death and unfavourable neurological outcomes in patients with moderate-to-severe TBI (GCS 3–12) [41]. Incorporating these molecular indicators into established prognostic models significantly improves predictive accuracy for mortality and severe disability, highlighting the potential of early biomarker profiling to refine and complement traditional clinical prognostication. Among other well-established prognostic markers, S100B has been shown to display a robust association between serum concentrations exceeding 0.1 μg/L at admission and poor neurological outcomes or increased mortality, particularly in severe TBI. Similarly, NSE, a marker of neuronal necrosis, correlates with prolonged coma duration and the development of vegetative states [42,43]. More recently, systemic and metabolic indices—such as the aspartate aminotransferase-to-platelet ratio index (APRI) and serum sirtuin 6 (SIRT6)—have emerged as novel prognostic factors, reflecting the interplay among neuroinflammation, oxidative stress, and systemic physiological responses to TBI. Integrative multivariate models combining GFAP, S100B, and NfL have demonstrated superior predictive performance for long-term outcomes, including the Glasgow Outcome Scale (GOS) and extended measures of neurocognitive recovery [44]. In a multicohort cross-sectional study, Edwards et al. reported that higher serum concentrations of GFAP, NfL, and tau were consistently associated with poorer neurobehavioral performance across the spectrum of mild, moderate, and severe TBI [45]. Collectively, these findings underscore the potential of circulating biomarkers as objective indicators of injury severity, neurobehavioral impairment, and long-term functional prognosis, reinforcing their role as essential components of precision neurotrauma care.

### 2.3. Monitoring Role

Monitoring biomarkers provide dynamic, longitudinal insights into the evolution of secondary injury and the response to therapeutic interventions. They are particularly valuable in intensive care settings, where continuous or serial evaluation of ongoing neurodegenerative processes can guide timely therapeutic decisions. Among these, GFAP and NfL have emerged as the most informative monitoring biomarkers: persistent elevations or secondary increases in NfL indicate progressive axonal degeneration, whereas declining GFAP concentrations are typically associated with stabilization or early neurological recovery [26,46]. In parallel, inflammatory cytokines such as IL-6, TNF-α, and interleukin-1β (IL-1β) serve as surrogate indicators of neuroinflammatory activity and secondary injury cascades [18]. Serial biomarker measurement can complement conventional physiological parameters, such as intracranial pressure (ICP) monitoring, providing a less invasive yet biologically sensitive approach to tracking disease dynamics over time. Emerging biosensor technologies and microfluidic platforms are poised to transform this field by enabling real-time, point-of-care biomarker detection. Such innovations hold the potential to redefine bedside neurocritical care, allowing for continuous molecular surveillance of TBI and personalized therapeutic modulation throughout the acute and subacute phases of TBI [47].

### 2.4. Therapeutic Role

Therapeutic biomarkers capture the biological response to specific interventions and constitute a fundamental element of personalized medicine approaches in TBI. They provide critical feedback on target engagement, dose optimization, and treatment efficacy, thereby enabling evidence-based therapeutic adjustment. For instance, SIRT6—a regulator of chromatin remodeling and DNA repair—has shown promise as both a prognostic and therapeutic biomarker: increased expression appears to promote neuronal survival and attenuate post-traumatic inflammation [44]. Likewise, inflammatory mediators such as IL-6 and TNF-α tend to decline in patients responding favorably to corticosteroid or neuroprotective therapies, reflecting modulation of the underlying inflammatory cascade. Metabolic and oxidative stress biomarkers, including the lactate/pyruvate ratio and F2-isoprostanes, are also being explored as indicators of mitochondrial-targeted interventions [48]. As novel therapeutic strategies—such as stem cell therapy, therapeutic hypothermia, and SIRT activators—advance through clinical trials, therapeutic biomarkers are poised to play an increasingly central role within adaptive and precision-based study designs. These molecular indicators will support individualized treatment selection and provide objective evaluation of neuroprotective efficacy, ultimately accelerating the personalization of care and improving outcomes for patients with TBI. The principal biomarkers currently employed in TBI are summarized in Table 1, Table 2, Table 3, Table 4, Table 5 and Table 6.

**Table 1:** Astroglial injury biomarkers (GFAP, S100B).

Biomarker	Key Studies	Type of TBI	Clinical Role	Sensitivity/Specificity/AUC	Notes
GFAP	Jalali et al. 2025 [32]; Bazarian et al. 2018 (ALERT-TBI) [38]; Papa et al. 2016 [49]; Korley et al. 2022 [41] (TRACK-TBI); Kaaber et al. 2024 [35]; Oris et al. 2024 [29]	Mild–severe	Rule-out CT injury; severity assessment; prognosis	Sens. 83–100%; Spec. 29–36%; AUC 0.73–0.94	Best performer for mild TBI; FDA-cleared (with UCH-L1) for CT rule-out
S100B	Oris et al. 2023 [39]; Park et al. 2018 [43]	Mild; Pediatric	Rule-out CT; early detection	Variable; high NPV	Widely used in Europe; extracranial release may reduce specificity

**Table 2:** Axonal injury biomarkers (Uch-L1, NfL, Tau).

Biomarker	Key Studies	Type of TBI	Clinical Role	Sensitivity/Specificity/AUC	Notes
UCH-L1	Jalali et al. 2025 [32]; Bazarian et al. 2018 [38]; Papa et al. 2016 [49]; Oris et al. 2024 [29]; Bishop et al. 2016 [34]	Mild–moderate	Diagnosis; CT rule-out when combined with GFAP	Sens. up to 97.6% (combined test); AUC 0.30–0.67	FDA-cleared only in combination with GFAP
NfL	Kaaber 2024 [35]; Edwards 2023 [45]	Mild–severe	Prognosis; persistent symptoms	AUC ~0.88 (TRACK-TBI)	Best long-term marker of axonal injury
Tau	Kaaber et al. 2024 [35]; Edwards et al. 2023 [45]	Mild–severe	Prognosis	Limited for diagnosis	Useful in repetitive TBI/chronic pathology

**Table 3:** Neuronal injury biomarkers (NSE).

Biomarker	Key Studies	Type of TBI	Clinical Role	Sensitivity/Specificity/AUC	Notes
NSE	Park et al. 2018 [43]; Haque et al. 2016 [50]	Mild–severe; Spinal cord	Early neuronal injury	Variable	Strongly affected by hemolysis

**Table 4:** Inflammatory biomarkers (IL-6, SIRT6, panels).

Biomarker	Key Studies	Type of TBI	Clinical Role	Sensitivity/Specificity/AUC	Notes
IL-6	Park et al. 2018 [43]	Pediatric	Early inflammation	NR	Elevation correlates with injury severity
SIRT6	Deping et al. 2025 [44]	Early TBI	Early prognosis	NR	Emerging predictive biomarker
Inflammatory panels	Clarke et al. 2024 [51]	Mild (persistent symptoms)	Symptom persistence profiling	NR	Combined CNS injury and inflammatory markers

**Table 5:** Genetic/risk biomarkers.

Biomarker	Key Studies	Type of TBI	Clinical Role	Notes
ApoE ε4 genotype	Merritt et al. 2018 [52]	Mild–moderate (veterans)	Risk of persistent symptoms; outcome prediction	Associated with poorer recovery

**Table 6:** Reviews providing synthetic evidence.

Study	Biomarkers Covered	Scope
Slavoaca et al. 2020 [30]	GFAP, UCH-L1, S100B, NSE, NfL	Comprehensive review
Najem et al. 2018 [31]	Classification, models, biomarkers	Mechanistic & methodological review
Abdelhak et al. 2022 [33]	GFAP	Biomarker-specific review
Bishop et al. 2016 [34]	UCH-L1	Structural and functional review
Hossain et al. 2024 [27]	Multiple biomarkers	Broad review
Hier et al. 2021 [42]	Mild TBI biomarkers	Clinical review
Oris et al. 2024 [29]	GFAP, UCH-L1, S100B	Mild TBI-focused review
Ghaith et al. 2022 [26]	Multiple biomarkers	Translational review

## 3. Types of Biomarkers

Biomarkers in TBI comprise a diverse array of molecular and physiological indicators that reflect distinct aspects of neuronal and glial pathology. They can be categorized into protein-based, genomic and transcriptomic, metabolomic and lipidomic, and imaging biomarkers, each offering complementary insights into brain injury mechanisms, disease progression, and therapeutic response [16].

### 3.1. Protein-Based Biomarkers

Protein biomarkers represent the most extensively investigated and clinically validated category in TBI research. They are measurable in blood, CSF, or other body fluids and reflect acute structural and metabolic disruptions of brain cells. Among these, GFAP and UCH-L1 are the leading candidates. GFAP, an astrocytic intermediate filament protein, is released following glial injury and blood–brain barrier (BBB) disruption, whereas UCH-L1 is a neuronal enzyme associated with axonal degeneration and synaptic dysfunction [49]. Their combined assessment improves diagnostic accuracy in mild TBI, achieving AUC values exceeding 0.93 for the detection of CT-positive lesions [35]. These biomarkers were the first to receive FDA approval for clinical application in 2018 as part of the Banyan Brain Trauma Indicator™ [53]. S100B, a calcium-binding protein secreted by astrocytes, is another well-established marker routinely used in European emergency departments. When measured within six hours of injury, S100B levels below 0.10 μg/L can reliably exclude the presence of intracranial pathology [42]. Additional protein biomarkers of note include NSE, which reflects neuronal necrosis, and tau and NfL, both indicative of axonal injury [45,50]. Although protein biomarkers have achieved the highest degree of clinical translation, several challenges remain. Many are influenced by extracranial injuries, renal clearance, and sampling timing. Consequently, integrating multiple protein markers with demographic and imaging data may enhance their clinical reliability and translational robustness [39].

### 3.2. Genomic and Transcriptomic Biomarkers

Genomic and transcriptomic biomarkers explore individual genetic susceptibility to TBI and the molecular responses triggered by injury. Advances in high-throughput sequencing have enabled the identification of single nucleotide polymorphisms (SNPs), gene expression profiles, and regulatory RNA molecules associated with injury severity and recovery outcomes [54]. Specific SNPs in genes encoding inflammatory cytokines (e.g., IL1B, TNF, APOE, BDNF) have been linked to prolonged symptoms and post-concussive syndrome [51]. For example, the APOE ε4 allele is associated with poorer cognitive recovery and an increased risk of chronic traumatic encephalopathy [52]. Equally relevant are microRNAs (miRNAs), small non-coding RNAs that regulate post-transcriptional gene expression. Altered expression of miR-21, miR-92a, and miR-425 has been observed within hours after injury, reflecting neuronal apoptosis and neuroinflammation [14,55]. Circulating miRNA panels are emerging as minimally invasive biomarkers for early diagnosis and prognosis, with some studies reporting AUC values exceeding 0.90 in differentiating TBI patients from healthy controls [56]. Moreover, transcriptomic analyses have revealed dynamic changes in the expression of genes involved in oxidative stress, synaptic repair, and neuroinflammation, highlighting potential targets for neuroprotective therapies [57]. Genomic and transcriptomic biomarkers thus hold substantial promise for precision medicine, enabling the identification of patients most likely to benefit from tailored therapeutic interventions.

### 3.3. Metabolomic and Lipidomic Biomarkers

Metabolomics and lipidomics represent emerging, systems-level approaches that profile small-molecule metabolites and lipid species altered after TBI. Because metabolism rapidly reflects changes in cellular homeostasis, these biomarkers capture early perturbations in energy metabolism, oxidative stress, and mitochondrial dysfunction [48,58]. Following TBI, characteristic alterations are observed in metabolites such as lactate, pyruvate, glutamate, succinate, and N-acetylaspartate (NAA), reflecting impaired oxidative phosphorylation and excitotoxicity. Lipidomic studies have identified changes in phosphatidylcholine and sphingomyelin species associated with membrane degradation and neuroinflammation [59]. Recent metabolomic analyses using mass spectrometry have demonstrated that serum profiles of specific amino acids and lipids can discriminate mild TBI from controls, and several metabolomic markers correlate with cognitive recovery and neuropsychological performance at 6–12 months post-injury [60]. Although still in an early translational phase, metabolomic and lipidomic biomarkers offer dynamic snapshot of metabolic distress and may effectively complement protein assays by supporting personalized therapeutic strategies and monitoring neuroprotective efficacy [61].

Consistent with these findings, large-scale clinical studies confirm that acute TBI induces reproducible alterations in both polar metabolites and complex lipid species, and that metabolite panels correlate with injury severity, neuroimaging features, and functional outcomes. A multicenter serum metabolomics study involving several hundred patients identified metabolite clusters and lipid signatures associated with TBI severity and prognosis, underscoring the translational potential of metabolomic biomarkers for early patient stratification [60]. High-resolution mass spectrometry (HRMS) platforms—including untargeted LC-MS workflows and targeted multiple-reaction-monitoring assays—have been central to the discovery and verification of small-molecule and lipid biomarkers. These techniques provide extensive coverage of glycerophospholipids, sphingolipids, fatty acids, and oxidized lipid species that reflect membrane breakdown and neuroinflammation. Several preclinical and translational studies have demonstrated that acute serum lipidome changes track with brain tissue alterations and with diffusion MRI markers of axonal injury, supporting the concept that circulating lipids may serve as peripheral surrogates of CNS pathology [61]. Longitudinal metabolomic profiling further reveals dynamic shifts in energy metabolites (e.g., lactate/pyruvate ratios and TCA cycle intermediates), amino acids, and lipid-remodeling pathways over days after injury. These temporal signatures help distinguish progressive secondary injury from early stabilization and have enabled the development of predictive outcome models when integrated with clinical variables. Notably, multiple studies applying machine-learning algorithms and pathway-enrichment analyses to metabolomic datasets have derived compact biomarker panels that outperform single-analyte approaches for severity classification and outcome prediction [62]. From a translational perspective, mass spectrometry discovery workflows are increasingly being complemented by targeted assay development (e.g., LC-MS/MS panels and immunoassays) designed to deliver reproducible and clinically actionable readouts. Methodological reviews emphasize best practices for sample handling, quality control, and study design, all of which are essential for progressing metabolomic and lipidomic candidates from discovery to validation and eventual clinical application. Furthermore, integrative multi-omics strategies—combining lipidomics, proteomics, and transcriptomics—have demonstrated superior diagnostic and prognostic performance compared with single-modality approaches, supporting the future development of multimodal molecular biomarker panels in TBI care [63]. Recent high-impact studies have provided compelling clinical evidence supporting the diagnostic and prognostic utility of metabolomic profiling in severe TBI. Banoei et al. [62] demonstrated that circulating metabolomic signatures can accurately stratify injury severity and distinguish primary injury patterns from evolving secondary injury processes in severe TBI. Using advanced multivariate and machine-learning–based analyses, these studies identified metabolite panels related to energy metabolism, oxidative stress, amino acid turnover, and lipid remodeling that were strongly associated with intracranial pathology, clinical severity scores, and patient outcomes [64]. More recently, an expanded longitudinal cohort confirmed that dynamic metabolomic trajectories across the acute and subacute phases predict neurological outcome and mortality, further supporting the translational potential of metabolomics as a tool for severity assessment and real-time monitoring of secondary injury [65].

### 3.4. Imaging Biomarkers

Imaging biomarkers provide non-invasive, spatially resolved assessments of structural and functional brain damage. Although not biochemical, they play a pivotal role in validating molecular findings and informing prognosis. MRI-based techniques such as diffusion tensor imaging (DTI), susceptibility-weighted imaging (SWI), and functional MRI (fMRI) enable the detection of diffuse axonal injury and microstructural white matter abnormalities that are not visible on conventional CT [66] 59. Quantitative metrics such as fractional anisotropy (FA) and mean diffusivity (MD) correlate with NfL levels and cognitive outcomes [67]. PET imaging provides functional biomarkers of neuroinflammation and metabolism, using tracers such as [^18^F]-FDG for glucose utilization and [^11^C]-PK11195 for microglial activation. Hybrid PET/MRI systems are increasingly employed in longitudinal TBI studies to map neurodegenerative changes. Emerging connectome-based and AI-driven imaging biomarkers integrate large-scale MRI datasets with machine learning algorithms to predict clinical outcomes and identify latent injury patterns [68]. These digital biomarkers complement molecular assays, bridging the structural, metabolic, and biochemical dimensions of TBI. The integration of biochemical and imaging biomarkers within multimodal predictive frameworks represents the future of precision diagnostics and personalized rehabilitation in TBI care.

### 3.5. Alternative Biomarker

In addition to well-established proteins such as GFAP, UCH-L1, and NfL, emerging biomarkers may enhance the assessment of brain damage after trauma by providing insights into axonal injury, demyelination, neuroinflammation, and BBB integrity.

- Spectrin breakdown products (SBDP-145/150)/α-II spectrin α-II spectrin is a component of the neuronal and axonal cytoskeleton. Following TBI, it undergoes proteolytic degradation, generating fragments known as spectrin breakdown products (SBDPs), particularly at 145 and 150 kDa. These fragments have been proposed as markers of acute axonal and cytoskeletal damage. Studies in animal models have shown that both α-II spectrin and its breakdown products increase after TBI [69].
- Matrix metalloproteinases (MMPs) regulate extracellular matrix remodeling BBB permeability. Following trauma, increased MMP-9 levels can disrupt tight junctions and the vascular basement membrane, promoting leakage of brain proteins into the bloodstream and exacerbating cerebral edema. MMP-9 is therefore considered a potential marker of BBB disruption and post-traumatic vascular instability, with similar findings reported in studies of neuroinflammation and vascular damage [70,71].
- Endothelial adhesion molecules—VCAM-1/ICAM-1 VCAM-1 and ICAM-1 are expressed on endothelial cells in response to inflammation or vascular stress. Elevated levels of these molecules may indicate endothelial activation, neurovascular inflammation, and potential compromise of the blood–brain barrier, all of which are relevant to brain trauma, long-term outcomes, and secondary neurodegenerative processes [72]. After head trauma, ICAM-1 expression on brain endothelium increases rapidly and remains elevated for an extended period. This upregulation contributes to neuroinflammation, leukocyte migration, and BBB impairment. In mouse models, genetic deletion of ICAM-1 (ICAM-1^−/−^) protects the brain from trauma, reducing leukocyte adhesion, preserving the BBB, and improving functional recovery [73]. Conversely, ICAM-1 activation after TBI is associated with cell death and behavioral deficits [72]. In controlled cortical impact models (CCI-TBI), treatment with anti-ICAM-1 agents reduces oxidative stress and neuropathology [74].
- GFAP breakdown products (GFAP-BDP) In addition to measuring intact GFAP, assessing its degradation fragments can provide additional information on the extent of astrocytic damage and glial remodeling after injury. GFAP-BDP may enhance sensitivity for detecting mild or diffuse astrocytic lesions that are not apparent on conventional imaging. Animal studies have shown that, after penetrating TBI, both total GFAP and GFAP-BDP levels remain elevated in brain tissue for up to three months. Furthermore, SBDP-145/150 levels increase in CSF and brain tissue from 24 h to seven days post-injury, suggesting that SBDP-145/150 may serve as a potential marker for the acute to subacute phase of TBI [75].
- The combined use of these biomarkers, even as a multimarker panel, could improve the sensitivity and specificity of diagnosing various forms of brain damage, including axonal injury, demyelination, vascular damage, and neuroinflammation. Integrating MBP, SBDP, MMPs, adhesion molecules, and GFAP-BDP with established markers (GFAP, UCH-L1, NfL) appears particularly promising in complex scenarios such as repeated trauma, diffuse injuries, and long-term follow-up. However, several limitations remain. Currently, many of these molecules lack validated clinical cut-offs, and much of the available evidence comes from preclinical models or small-scale studies. Variability in analytical methods, sampling timing, clearance rates, and protein degradation complicates standardization for routine clinical application.

## 4. Clinical Utility and Limitations

The translation of TBI biomarkers from experimental discovery to clinical implementation represents a significant milestone in neurotrauma research. Biomarkers such as GFAP, UCH-L1, S100B, and NfL have demonstrated robust diagnostic and prognostic performance, complementing traditional neuroimaging and clinical scoring systems [16]. Nevertheless, challenges related to assay variability, sampling timing, and methodological standardization continue to limit their widespread adoption in routine practice. Biomarkers offer clear advantages over subjective neurological assessments in the acute phase of injury. In the multicentre TRACK-TBI and CENTER-TBI cohorts, the combined measurement of GFAP and UCH-L1 achieved a sensitivity of 97.6% and a specificity of 99.6% for detecting CT-positive lesions within 12 h of injury. In contrast, S100B shows a sensitivity of approximately 90–95% but lower specificity (40–60%), particularly in polytrauma patients, due to its extracranial expression in muscle and adipose tissue [42]. NfL, an axonal structural protein, is strongly associated with diffuse axonal injury (DAI) and long-term neurocognitive outcomes. Serum NfL levels correlate closely with diffusion tensor imaging (DTI) abnormalities and with the severity of TBI. Both NfL and GFAP are predictive of intracranial abnormalities; however, only GFAP has been consistently linked to poor outcomes at six months post-injury [76]. The temporal dynamics of biomarker release after injury are highly variable and biomarker-specific. In a large cohort of trauma patients, GFAP became detectable within one hour of injury and peaked around 20 h, whereas UCH-L1 peaked much earlier—around 8 h post-injury—and declined rapidly over the next 48 h [49]. This variability implies that the timing of blood sampling relative to the moment of trauma substantially influences measured concentrations and diagnostic yield. A recent review emphasized optimal sampling windows: S100B within 0–4 h, UCH-L1 (and tau) within 4–12 h, GFAP between 12–36 h, and NfL potentially only after several days [42]. While no single biomarker achieves perfect diagnostic accuracy, multimarker panels—such as GFAP + UCH-L1 + NfL—significantly enhance predictive performance, particularly when integrated with clinical and imaging data. These approaches represent a paradigm shift toward integrated, multimodal assessment rather than reliance on isolated markers. Neuroimaging and neurological scores remain the gold standards for TBI evaluation, yet they have intrinsic limitations. Computed tomography enables rapid detection of hemorrhage and mass effect but lacks sensitivity for microstructural and metabolic injury. Magnetic resonance imaging and diffusion tensor imaging (DTI) improve detection of diffuse axonal injury but are costly, time-consuming, and often impractical in emergency settings [66]. The Glasgow Coma Scale (GCS), a cornerstone of neurological assessment, provides valuable functional information but is inherently subjective and can be confounded by sedation, intoxication, or systemic factors [77]. In contrast, biomarkers offer objective, quantifiable, and reproducible data unaffected by these confounders. For mild TBI, combining biomarkers with imaging substantially improves sensitivity. Studies have shown that GFAP levels above 286 pg/mL can detect CT-positive lesions even in patients with normal GCS scores [49]. In moderate-to-severe cases, integrating S100B and NfL with MRI or GCS data enhances prognostic accuracy for long-term cognitive and functional recovery [27]. Thus, biomarker-based assessment does not replace imaging or clinical evaluation but augments diagnostic precision and enables effective risk stratification, particularly in resource-limited or prehospital settings. Despite substantial progress, several limitations continue to hinder the full clinical integration of TBI biomarkers. A first set of challenges relates to biological and preanalytical variability. Biomarker levels may vary with age, sex, comorbidities, extracranial injuries, and renal function. For example, S100B levels can increase after bone fractures or soft tissue trauma, leading to false-positive results [21]. Similarly, preanalytical variables—such as sample handling, centrifugation time, and storage conditions—can substantially influence measured concentrations. Different analytical platforms (ELISA, Simoa, ECLIA) and reagent batches may also produce variable results, limiting inter-laboratory comparability [66]. Moreover, many biomarkers—particularly S100B and NSE—are not brain-specific, and cross-reactivity with peripheral tissues can reduce specificity, especially in polytrauma or systemic inflammatory conditions. These sources of variability underscore the need for CNS-specific isoforms or post-translational modifications to enhance biomarker selectivity [18]. A second major limitation concerns the temporal dynamics of biomarker release. Each marker exhibits a distinct kinetic profile: UCH-L1 peaks within six hours and declines rapidly, whereas GFAP and NfL remain elevated for days to weeks [37,78]. This heterogeneity complicates result interpretation when sampling is not synchronized with the underlying biological time-course, contributing to between-study discrepancies and uncertainties in defining reliable clinical thresholds. Real-world sampling considerations extend well beyond the biological time-course. Factors such as patient transport speed, laboratory availability, sample handling conditions, and sampling frequency can substantially influence measurement reliability. In acute trauma or prehospital settings, ensuring optimal sampling is often challenging, which may limit diagnostic sensitivity and specificity and reduce the clinical utility of biomarker panels. Integration with imaging techniques (CT, MRI/DTI) and clinical scores (GCS) remains essential. Biomarkers provide insights into biological and molecular aspects of brain injury—such as astrocytic activation, axonal damage, and microstructural disruption—that may not immediately correspond to CT findings or visible neurological changes. However, the diagnostic and prognostic value of this multimodal approach depends critically on appropriate temporal alignment: collecting samples too early or too late can weaken correlations with radiological abnormalities or clinical outcomes. For example, in chronic or subacute TBI, NfL may remain significantly elevated even 30 days post-injury, reflecting ongoing axonal degeneration. This suggests that NfL is valuable for longitudinal monitoring and prognostication but should not replace imaging or comprehensive clinical assessment [79]. Thus, temporal, sampling, and multimodal factors collectively influence not only acute diagnosis but also long-term monitoring and prognosis. International initiatives such as the BIOSIGNAL and CENTER-TBI biomarker consortia are working to harmonize protocols and establish standardized reference ranges. Although the FDA has approved GFAP and UCH-L1 for clinical use, most biomarkers remain confined to research settings. Regulatory approval requires multicentre validation, cost-effectiveness analyses, and demonstration of measurable clinical impact—processes that are both time- and resource-intensive [44]. Without standardized protocols defining when, how, and how often samples should be collected—and how biomarker data should be integrated with imaging and clinical information—clinical translation will remain incomplete. Future research must therefore establish clear operational procedures, optimal temporal windows, and integrated multimodal strategies to enable effective real-world implementation.

## 5. Influence of BBB Integrity, Injury Mechanism, and Confounders on Biomarker Utility

Beyond technical and analytical limitations, the clinical performance of TBI biomarkers is also shaped by pathophysiological and contextual factors that determine their diagnostic and prognostic utility. BBB integrity plays a central role: biomarkers such as GFAP, UCH-L1, and S100B enter the circulation primarily following BBB disruption. Limited BBB permeability—as often occurs in mild or diffuse injuries—can delay or attenuate measurable concentrations [80]. Conversely, extensive BBB damage in moderate-to-severe TBI or hemorrhagic lesions results in rapid biomarker release and higher early diagnostic yield. The mechanism of injury also influences biomarker patterns: GFAP is typically elevated in focal injuries involving astroglial disruption, whereas NfL is more strongly associated with diffuse axonal injury, blast exposure, and rotational acceleration forces [81,82]. This differentiation supports their complementary value within multimarker diagnostic panels. Several confounders—including extracranial trauma (affecting S100B), renal dysfunction (affecting biomarker clearance), age-related baseline variability, and systemic inflammation—can alter biomarker levels and must be considered when interpreting results [4,83]. Understanding how BBB integrity, injury mechanism, timing, and confounders modulate biomarker profiles is therefore essential to maximizing their real-world clinical utility and ensuring accurate interpretation across heterogeneous TBI populations.

## 6. Materials and Methods

We conducted a comprehensive literature search and review of studies published in the last 10 years to identify clinical, pathophysiological, and biomarker studies on TBI. PubMed was the primary database searched using the following keyword clusters and synonyms, combined with Boolean operators: (“traumatic brain injury” OR “TBI” OR “mTBI” OR “mild traumatic brain injury”) AND (“clinical features” OR “clinical presentation” OR “symptoms”) OR (“pathophysiology” OR “mechanism*” OR “biomechanics*”) OR (“dementia” OR “neurodegeneration”) OR (“biomarker*” OR “GFAP” OR “glial fibrillary acidic protein” OR “UCH-L1” OR “UCHL1” OR “prognostic biomarker*” OR “prognosis”) OR (“inflamm*” OR “neuroinflammation” OR “blood-brain barrier”). Search terms were adapted to PubMed syntax where appropriate, with filters applied to restrict results to human studies and English-language publications. Reference lists of included articles and recent reviews were also screened to identify additional relevant publications. All retrieved records were imported into Mendeley for deduplication and initial organization. Following deduplication, titles and abstracts were independently screened by two reviewers against pre-specified inclusion and exclusion criteria. Inclusion criteria comprised: original research articles, systematic reviews or meta-analyses, and clinical or translational studies addressing TBI clinical features, pathophysiology, neuroinflammation, blood–brain barrier disruption, TBI-related dementia risk, or biomarkers (including GFAP and UCH-L1), published within the specified 10-year timeframe. Exclusion criteria included: non-English publications, animal-only studies (unless providing translational biomarker or pathophysiology data explicitly discussed in a clinical context), conference abstracts without full text, case reports with fewer than three patients, and articles older than 10 years.

## 7. State of the Art and Future Direction

This review highlights that biomarkers represent one of the most promising frontiers for improving TBI diagnosis, prognosis, and clinical management [84]. Despite remarkable technological and methodological advances over the past two decades, the biological and clinical complexity of TBI continues to challenge precision medicine approaches [85]. The integration of molecular, clinical, and neuroimaging parameters is now widely recognized as the most effective strategy to overcome limitations of traditional assessment tools—such as the GCS and CT—which, while essential, often fail to capture the microstructural and dynamic nature of brain injury. Protein biomarkers, particularly GFAP and UCH-L1, have achieved sufficient clinical validation to enable routine use in acute care settings, as evidenced by FDA approval of the Banyan Brain Trauma Indicator™ [86]. Their high sensitivity and specificity for detecting clinically significant intracranial lesions represent a major advance, especially in mild TBI where diagnosis is often hindered by absent radiological findings [87]. However, interindividual variability, differences in release and clearance kinetics, and concerns regarding tissue specificity remain significant limitations. Emerging biomarkers such as NfL, S100B, tau, and NSE provide valuable insights into axonal injury and post-traumatic neurodegeneration, supporting a pathophysiological continuum between acute TBI and chronic neurodegenerative disorders, including Alzheimer’s disease and chronic traumatic encephalopathy (CTE). This connection underscores TBI not merely as an isolated event but as a potential trigger for long-lasting neuropathological processes driven by chronic inflammation, oxidative stress, and mitochondrial dysfunction [88]. Beyond protein markers, genomic, transcriptomic, and metabolomic approaches are reshaping our systemic understanding of TBI. Altered circulating microRNA profiles and metabolic signatures offer dynamic snapshots of early cellular responses and recovery trajectories, paving the way for precision medicine based on individualized molecular profiles [89]. Nevertheless, clinical translation of these omics-based strategies remains nascent, hindered by high costs, methodological heterogeneity, and the need for large-scale multicentre validation. A critical barrier across studies is the lack of standardization in sampling protocols, sample handling, analytical platforms, and data interpretation, which impedes result comparability and establishment of universal diagnostic thresholds. The most promising clinical direction involves multimodal assessment strategies combining biochemical markers, advanced neuroimaging (e.g., DTI, fMRI, PET), and artificial intelligence–based predictive models to stratify risk, monitor injury progression, and tailor therapeutic interventions. Emerging miniaturized biosensors and microfluidic platforms may soon enable real-time molecular monitoring, potentially transforming TBI management in emergency and critical care settings. Despite these advances, several challenges persist, and this review has intrinsic limitations. To date, no biomarker demonstrates complete brain-tissue specificity, leaving unresolved the challenge of correlating circulating concentrations with precise injury location and extent. It is essential to distinguish between absolute tissue specificity and practical diagnostic utility when interpreting circulating TBI biomarkers. While no currently available biomarker is exclusively expressed in brain tissue, several—including GFAP, UCH-L1, and NfL—exhibit marked CNS enrichment and have demonstrated robust diagnostic and prognostic performance in large clinical validation studies. Thus, despite limited extracranial expression under specific conditions, these biomarkers retain high practical specificity for brain injury in most clinical contexts. This distinction is particularly relevant in polytrauma settings, where extracranial contributions may modestly influence circulating levels but do not negate their overall diagnostic value in TBI. The clinical relevance of this concept is underscored by regulatory validation: GFAP and UCH-L1 are components of the FDA-cleared Banyan Brain Trauma Indicator™ for evaluating mild TBI in adults. This supports the principle that, despite lacking absolute tissue exclusivity, these biomarkers possess enough practical diagnostic specificity to guide clinical decision-making in real-world TBI settings [90]. A major unresolved challenge in metabolomic biomarker research for TBI is distinguishing CNS-specific metabolic signals from systemic metabolic responses to trauma. Many metabolites detected in peripheral blood after injury reflect global physiological stress responses—including hypoxia, mitochondrial dysfunction, systemic inflammation, catecholamine surge, and multi-organ injury—rather than brain-derived pathology per se. Consequently, metabolomic signatures may capture systemic trauma severity as much as, or more than, CNS damage. Several methodological strategies are therefore required to enhance CNS specificity: inclusion of extracranial trauma control cohorts, parallel cerebrospinal fluid–plasma profiling, multimodal correlation with neuroimaging and validated protein biomarkers, and longitudinal within-subject analyses to differentiate primary brain injury from secondary systemic effects. Failure to address these confounders risks overestimating the brain specificity and clinical interpretability of circulating metabolomic biomarkers in TBI. Moreover, the limited number of large-scale longitudinal studies restricts our understanding of long-term outcomes. Ethical and regulatory considerations surrounding large-scale omics-derived data use also warrant careful attention, particularly regarding data security, patient consent, and clinical decision-making. Future research should prioritize multicentre studies to harmonize sampling and analytical procedures. Additionally, developing and validating composite clinical scores that integrate symptoms, neuroimaging findings, and biomarker profiles would represent a decisive step toward truly multimodal TBI management. Biomarkers are reshaping the TBI care paradigm, enabling a transition from reactive to predictive, personalized intervention models. Although full clinical integration remains ongoing, the growing evidence base and rapid technological advances suggest a future where TBI diagnosis and treatment will be guided not only by clinical and radiological parameters but also by deeper insights into underlying molecular mechanisms. A major barrier to clinical translation of both protein and metabolite biomarkers in TBI is the need for rigorous analytical and clinical validation. Reliable implementation requires replication across large, independent, demographically diverse cohorts, together with strict standardization of pre-analytical variables—including sample collection, processing, storage conditions, and timing relative to injury. Analytical reproducibility across platforms and laboratories is also essential, particularly for metabolomics where inter-platform variability and batch effects can substantially influence results. Beyond analytical robustness, biomarker performance must be evaluated within integrated clinical models incorporating injury mechanism, neuroimaging, physiological variables, and longitudinal outcome measures. Without harmonized analytical pipelines and multi-cohort validation, biomarker signatures—especially complex metabolomic profiles—remain susceptible to cohort-specific bias and limited generalizability, constraining their translation into routine clinical practice. In conclusion, this review distinguishes itself from previous publications on TBI biomarkers through its comprehensive, up-to-date synthesis that systematically integrates proteomics, metabolomics, lipidomics, genomics, advanced neuroimaging, and emerging diagnostic technologies. Unlike most existing literature, which primarily focuses on traditional protein biomarkers, this work expands the scope to include metabolic and lipidomic signatures, as well as genomic and transcriptomic markers such as microRNAs and SNPs associated with injury vulnerability. It also addresses emerging indicators of cytoskeletal damage, vascular dysfunction, and neuroinflammation—including SBDP-145/150, GFAP-BDP, MMP-9, ICAM-1, and VCAM-1. Furthermore, this review provides an updated discussion of new analytical platforms and detection methods—such as high-resolution mass spectrometry, targeted LC-MS/MS, microfluidics, and point-of-care biosensors—emphasizing their potential to enhance sensitivity, specificity, and diagnostic speed. An innovative contribution is the exploration of novel clinical applications, including individualized risk stratification, personalized prognostication, longitudinal monitoring of secondary injury, and integration with advanced neuroimaging (MRI, DTI, PET) and machine learning within multimodal predictive models. In parallel, this article offers a thorough critical analysis of persistent challenges hindering clinical translation, including confounding variables, pre-analytical variations, lack of validated cut-off values for emerging biomarkers, and absence of standardized protocols for sample collection and analysis. Collectively, these elements position this work as a significant contribution to the field, providing a comprehensive synthesis that not only summarizes current knowledge but also delineates the most promising directions for advancing molecular and multimodal diagnostics in TBI.

## 8. Conclusions

Biomarkers are emerging as essential tools for enhancing TBI diagnosis, prognosis, and longitudinal monitoring. However, no single marker has achieved sufficient specificity or standardization to supplant existing clinical and radiological methods. The most promising direction involves multimodal platforms integrating biochemical biomarkers, advanced neuroimaging, and machine learning–based predictive models to comprehensively capture TBI heterogeneity and enable individualized patient management.

## Data Availability

No new data were created or analyzed in this study. Data sharing is not applicable to this article.

## Abbreviations

The following abbreviations are used in this manuscript:

AD	Alzheimer’s Disease
AI	Artificial Intelligence
AUC	Area Under the Curve
BBB	Blood–Brain Barrier
BDNF	Brain-Derived Neurotrophic Factor
CTE	Chronic Traumatic Encephalopathy
CSF	Cerebrospinal Fluid
CNS	central nervous system
CT	Computed Tomography
DAI	Diffuse Axonal Injury
DTI	Diffusion Tensor Imaging
ELISA	Enzyme-Linked Immunosorbent Assay
ECLIA	Electrochemiluminescence Immunoassay
FDA	Food and Drug Administration
FA	Fractional Anisotropy
FDG	Fluorodeoxyglucose (PET tracer)
fMRI	Functional Magnetic Resonance Imaging
GCS	Glasgow Coma Scale
GFAP	Glial Fibrillary Acidic Protein
GOS	Glasgow Outcome Scale
ICP	Intracranial Pressure
ICU	Intensive Care Unit
IL	Interleukin
IL-1β	Interleukin-1 beta
IL-6	Interleukin-6
IL1B	Interleukin-1 beta gene
miRNA	MicroRNA
MD	Mean Diffusivity
MRI	Magnetic Resonance Imaging
mTBI	Mild Traumatic Brain Injury
NAA	N-Acetylaspartate
NF-L/NfL	Neurofilament Light Chain
NIH	National Institutes of Health
NINDS	National Institute of Neurological Disorders and Stroke
NSE	Neuron-Specific Enolase
PET	Positron Emission Tomography
SNP	Single Nucleotide Polymorphism
S100B	(Already abbreviation—calcium-binding astrocytic protein)
SIRT6	Sirtuin 6
SWI	Susceptibility-Weighted Imaging
TBI	Traumatic Brain Injury
TNF-α	Tumor Necrosis Factor-alpha
UCH-L1	Ubiquitin Carboxy-Terminal Hydrolase L1
USD	United States Dollar
